# Differentially methylated and expressed genes in familial type 1 diabetes

**DOI:** 10.1038/s41598-022-15304-5

**Published:** 2022-06-30

**Authors:** Mohammed Dashti, Rasheeba Nizam, Prashantha Hebbar, Sindhu Jacob, Sumi Elsa John, Arshad Channanath, Hessa Al-Kandari, Thangavel Alphonse Thanaraj, Fahd Al-Mulla

**Affiliations:** 1 Department of Genetics and Bioinformatics, Dasman Diabetes Institute, Kuwait City, Kuwait; 2 Department of Population Health, Dasman Diabetes Institute, Kuwait City, Kuwait; 3 Department of Pediatrics, Farwaniya Hospital, Ministry of Health, Kuwait City, Kuwait

**Keywords:** Genetics, Immunology, Endocrinology

## Abstract

There has recently been a growing interest in examining the role of epigenetic modifications, such as DNA methylation, in the etiology of type 1 diabetes (T1D). This study aimed to delineate differences in methylation patterns between T1D-affected and healthy individuals by examining the genome-wide methylation of individuals from three Arab families from Kuwait with T1D-affected mono-/dizygotic twins and non-twinned siblings. Bisulfite sequencing of DNA from the peripheral blood of the affected and healthy individuals from each of the three families was performed. Methylation profiles of the affected individuals were compared to those of the healthy individuals Principal component analysis on the observed methylation profiling based on base-pair resolution clustered the T1D-affected twins together family-wide. The sites/regions that were differentially methylated between the T1D and healthy samples harbored 84 genes, of which 18 were known to be differentially methylated in T1D individuals compared to healthy individuals in publicly available gene expression data resources. We further validated two of the 18 genes—namely *ICA1* and *DRAM1* that were hypermethylated in T1D samples compared to healthy samples—for upregulation in T1D samples from an extended study cohort of familial T1D. The study confirmed that the *ICA1* and *DRAM1* genes are differentially expressed in T1D samples compared to healthy samples.

## Introduction

A dramatic increase in the incidence and prevalence of type 1 diabetes (T1D) has been witnessed in the past few decades. This poses a major challenge to the health care system worldwide. T1D accounts for 10% of the total cases of diabetes, with a global annual estimated incidence of 128,900 and prevalence of 1,110,100 in children under the age of 20 years^1^. Despite this, relatively little is known regarding the etiopathogenesis of the disease. T1D is defined as a lifelong autoimmune disease characterized by an immune response against pancreatic beta cells, resulting in little or no insulin production.

Multiple lines of evidence indicate the collective role of genetic, immunological, and environmental factors in the onset and progression of T1D. The genetic risk of T1D has been evidenced by concordance and heritability rates > 50% in monozygotic twins in long-term follow-up studies^2^. To date, more than 60 susceptibility gene loci have been implicated in the pathogenesis of T1D^3^. The human leukocyte antigen (HLA) region accounts for 50% of the genetic risk of T1D. Presence of specific HLA DR/DQ haplotypes tend to increase the susceptibility to T1D^4^. An autoimmune pathology of T1D is largely evidenced by a series of T and B cell-mediated events that lead to intolerance to self-antigens, resulting in chronic beta-cell inflammation. In support of this notion, the four major autoantibodies that characterize T1D include islet cell antibodies (ICA), antibodies to glutamic acid decarboxylase (GAD-65), insulin autoantibodies (IAA), and IA-2A, antibodies to protein tyrosine phosphatase^5^. Environmental factors, such as rapidly evolving lifestyle changes, viral infections, toxins, and dietary habits, are often implicated in the pathogenesis of T1D^6^; however, their specific roles have been poorly studied.

Literature reports indicate the need for deciphering the complex intertwining between the genetic and environmental architecture of T1D to get an improved knowledge of its onset and progression. The incomplete concordance of T1D incidence in monozygotic twins convincingly indicates the role of the gene-environment interactions and epigenetics in the pathogenesis of the disease^7^. Paul et al.^8^, by way of performing an epigenome-wide association study on 52 monozygotic twin pairs discordant for T1D, observed a substantial enrichment of differentially variable CpG positions in T1D twins when compared with their healthy co-twins or with unrelated healthy individuals. Stefan et al*.*^9^ observed significant differences in methylation patterns in known T1D-associated genes, such as HLA, INS, IL-2RB, and CD226, and further suggested that abnormalities in DNA methylation patterns in genome regions known to regulate gene transcription are possibly involved in the pathogenesis of T1D. In addition, Paul et al.^8^ found evidence even from cord blood of new-borns who progress to overt T1D to suggest that differentially variable CpG positions (DVP) among twins discordant for T1D likely emerge soon after birth. Johnson et al.^10^ substantiated the role of DNA methylation in T1D by establishing that methylation differences are present before both islet autoimmunity (IA) onset and diabetes diagnosis. The incidence and prevalence of T1D have been reported to be alarmingly high in the Arabian Peninsula possibly due to its unique genetic makeup, high rate of consanguinity (54.3%), extreme climatic conditions, and rapidly evolving lifestyle changes^11^. Familial aggregation of T1D is high among Arabs. Studies from Kuwait, which has a high rate of consanguineous marriages, have reported a familial form of T1D in 33% of patients with T1D and the incidence of T1D has doubled in Kuwaiti children in the last 20 years^12^. Perhaps, the gene-environment interactions and environmental conditions in the Arabian Peninsula are different from those in Europe where earlier methylation studies were mostly initiated. Hence, the present study aimed to understand the extent to which epigenetic factors contribute to T1D pathogenesis. We performed genome-wide methylation on a cohort consisting of three Arab families from Kuwait with mono-/dizygotic twins and non-twinned siblings concordant for T1D phenotype. Additionally, we integrated publicly available gene expression data with the methylation profile to explore the causality between DNA methylation and transcriptional activities and thereby to prioritize the candidate methylome markers obtained in this study.

## Results

### Clinical characteristics of the study cohort

The study cohort consisted of three families. There were at least two family members afflicted with T1D in each family (Table 1). The homozygosity between the two siblings in Family B and dizygosity between the two siblings in Family A were confirmed for genetic correlations by analysis of the whole exome sequence data for frequencies of heterozygous and homozygous exome variants (Supplementary Information). In addition, HLA typing profile of the T1D affected individuals from the three families (Supplementary Information) were positive for HLA-DRB1*03:01 in haplotypic association with DQA1*05:01-DQB1*02:01 indicating high risk of developing T1D^4^. The two children of Family C (who were aged 9 and 14 years at the time of recruitment) had their age at onset of T1D recorded as 2 and 1 years. Nevertheless, it is to be noted that they were diagnosed for T1D clinically and they tested positive for high-risk HLA DR/DQ haplotypes.

**Table 1:** Clinical characteristics of the study cohort.

Family	Members	Symbol	Phenotype	Age at recruitment (years)	Age at onset of T1D (years)
A	Father	I_1_A	healthy	46	–
	Mother	I_2_A	healthy	34	–
	Daughter	II_1_A	healthy	14	–
	Son	II_2_A	T1D	7	5
	Daughter *	II_3_A	T1D	12	6
	Daughter *	II_4_A	T1D	12	6
B	Mother	I_1_B	healthy	37	–
	Father	I_2_B	T1D	39	12
	Daughter	II_1_B	healthy	6	–
	Son	II_2_B	healthy	9	–
	Daughter	II_3_B	T1D	13	11
	Daughter †	II_4_B	T1D	11	10
	Daughter †	II_5_B	T1D	11	10
C	Father	I_1_C	healthy	43	–
	Mother	I_2_C	healthy	42	–
	Son	II_1_C	T1D	9	2
	Daughter	II_2_C	T1D	14	1

Twins; dizygotic *, monozygotic †.

### DNAm events across the three familial T1D

The targeted bisulfite sequencing facilitated deriving the DNAm profiling in peripheral blood samples of the individuals from the three families with T1D aggregation. Quality control metrics measured on the generated data (Supplementary Information) revealed (a) a consistent percentage methylation pattern which showed that bases were either methylated or unmethylated uniformly across all the samples (Supplementary Figure S1) and (b) a consistent read coverage per base of CpG sites featuring no significant difference among the samples (Supplementary Figure S2). These observations indicate that our experiments did not suffer from any bias secondary to PCR duplication. After fulfilling the criteria for calling DNAm events (Methods and Material section), the methylKit tool managed to identify 85,922 CpG single sites, 70,625 tiles, and 65,494 truseq regions across the three families.

**Figure 1:**
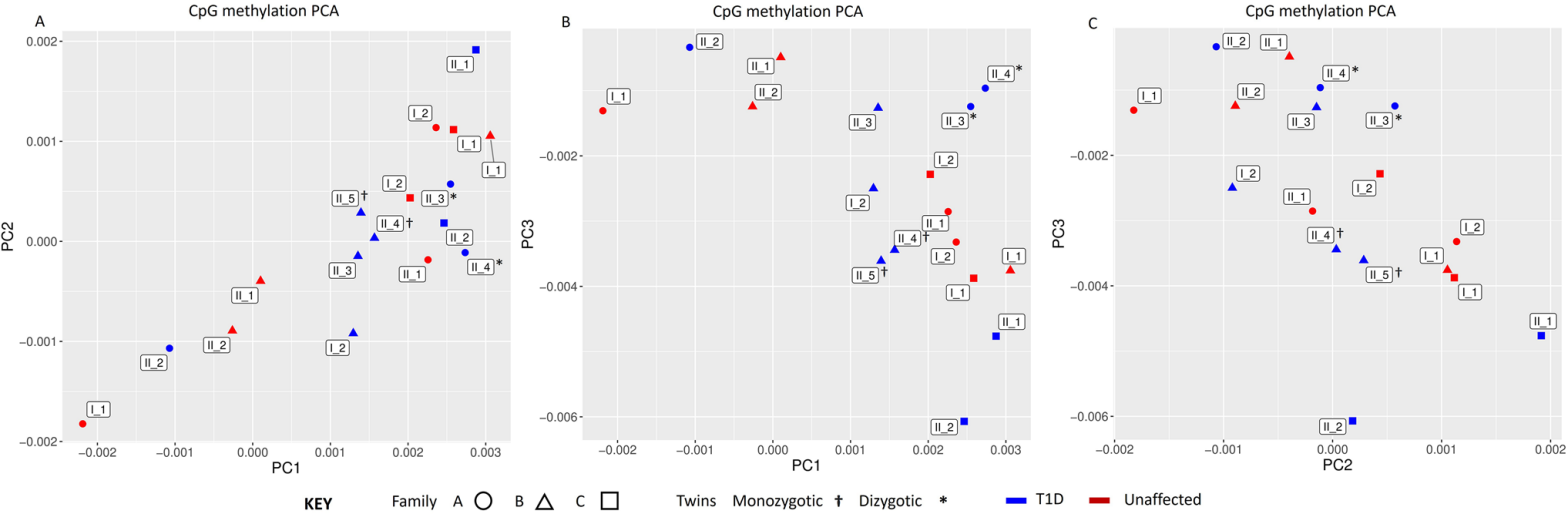
Illustration of inter-individual/family CpG methylome variation observed from PCA plot of the three T1D affected families. ‘I’ refers to parents; ‘II’ refers to children. Blue colour represents T1D individuals while red colour represents unaffected individuals. (**A**) depicts methylome differences observed among samples across the principal components of PC1-PC2; (**B**) depicts across the principal components of PC1-PC3; and (**C**) depicts across the principal components of PC2-PC3. The T1D individuals (in Blue) tend to cluster together, familywise. Similarly, monozygotic (†) and dizygotic (*) twins tend to cluster together suggesting a closer methylome architecture among them.

The screen plot of unsupervised PCA model of samples suggested that the principal component 1 (PC1) could explain 40% of the methylome variation observed (among samples), while the PC2 and PC3 could explain 34% and 32%, respectively (Supplementary Figure S3). Figure 1A–C presents the distribution of methylome variation observed among the individuals from the first three principal components. All the affected individuals clustered together familywise in one or the other of the PC1-PC2, PC2-PC3, and PC3-PC1 plots. Interestingly, the affected monozygotic (indicated by † in the plots) and dizygotic (indicated by * in the plots) twins clustered together in all the three PCs, suggesting a closer methylome architecture among twins. This observed close clustering of the twins is in line with our expectation, as twins share a common environmental exposure. It further rules out spurious methylation variation contributed by confounding factors.

### Differential DNAm events

The methylKit tool was able to detect, localize, and annotate differential DNAm events (differentially methylated CpG sites, DMC and differentially methylated regions, DMR) along with their methylation direction (hyper- or hypo- methylation in T1D versus healthy individuals) across the three T1D families (Supplementary Table S1).

Supplementary Figure S4 presents the results of the analysis for mapping the genome locations of the DMC and DMR through *genomation* tool. The results indicate that a majority of the observed differentially methylated events occurred at intronic and intergenic regions, rather than at promoter and exon regions. Similarly, differentially methylated CpG events were more prevalent outside of CpG islands and shore regions.

The observed DMC and DMR (tiles and truseq regions) mapped to 84 unique genes (Supplementary Table S2), including coding, non-coding, and uncharacterized genes, as well as microRNAs (miRNAs). None of these 84 genes overlapped with published T1D association signals in the GWAS Catalog (www.ebi.ac.uk/gwas/).

### Pathway enrichment analysis

**Figure 2:**
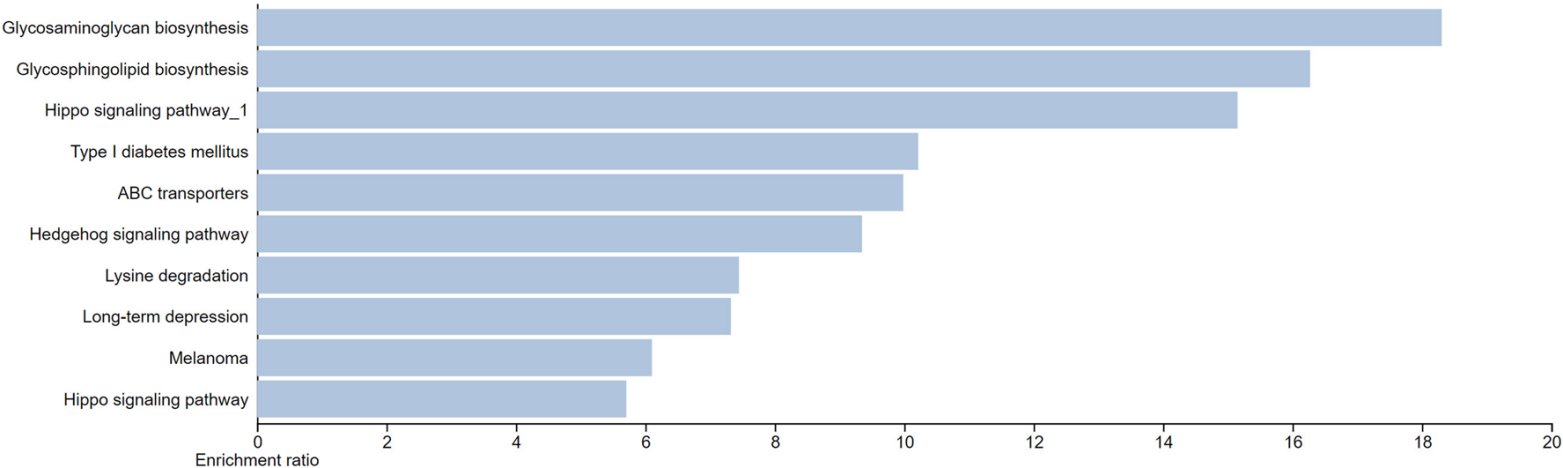
KEGG pathways identified as enriched by genes that are differentially methylated in T1D versus healthy individuals in our study. All the identified functional biological processes and pathways were in light blue indicating an FDR p-value of > 0.05.

Results of pathway enrichment analysis pointed to 10 pathways (including T1D pathways) that are enriched by the differentially methylated genes (Fig. 2). However, none of the identified pathways passed the significance level defined by an *FDR* of < 0.05. The inability to identify any significantly enriched pathway is probably because of the small size of the list of examined genes.

### Altered DNAm and differential expression of T1D genes—prioritizing candidate genes

Upon comparing the differentially methylated genes from our study against a list of published datasets of differentially expressed T1D genes of eight gene expression datasets (Supplementary Table S3), it was found that 18 of the 84 unique genes overlapped in both the datasets. These 18 genes (Table 2) could be considered as candidate genes that are more likely to be involved in the T1D pathogenesis. Functional regions that are differentially methylated from these genes consist of five CpG islands, 13 introns, eight shores, and six promoters, as well as exons. Of these 18 genes, Islet Cell Autoantigen 1 (*ICA1*) and Matrix Metallopeptidase 26 (*MMP26*) were significantly differentially methylated in DMC and DMR (tiles and truseq regions) analysis, while DNA Damage Regulated Autophagy Modulator 1 (*DRAM1*) and TIMP Metallopeptidase Inhibitor 3 (*TIMP3*) were significantly differentially methylated in DMR (tiles and truseq regions). Further, a subset of five differentially methylated genes, namely Ubiquitin Specific Peptidase 22 (*USP22*), Chaperonin Containing TCP1 Subunit 7 (*CCT7*), RAN Binding Protein 3 (*RANBP3*), Phosphatidylinositol-4,5-Bisphosphate 3-Kinase Catalytic Subunit Gamma (*PIK3CG*), and Protocadherin Gamma Subfamily B, 6 (*PCDHGB6*), overlapped with more than one T1D gene expression datasets that were from pancreatic and blood samples.

**Table 2:** Differentially methylated events across three familial T1D overlapped with differentially expressed genes of published T1D datasets.

Genes	Methylation direction	DMC	DMR (tiles,truseq regions)	Functional regions	Expression direction	Expression accession ID
*EXT1*	Hypo	8:119,080,372–119,080,372	-,-	intron	Control up vs T1D	GSE33440
*ICA1*	Hyper	7:8,171,773–8,171,773 7:8,171,803–8,171,803	7:8,171,001–8,172,000, 7:8,171,680–8,171,904	intron	Control down vs T1D clinical onset	E-MEXP-1140
*PIK3CG*	Hypo	7:106,513,011–106,513,011	-,-	exon	Control down vs T1D recent onset	E-MEXP-1140
					Control down vs T1D	GSE33440
*PCDHGB6*	Hypo	5:140,789,268–140,789,268	-,-	CpGi,exon,intron	Control up vs T1D clinical onset	E-MEXP-1140
					Control up vs T1D	GSE33440
*ST3GAL6*	Hyper	3:98,504,779–98,504,779	-,-	intron	Control up vs T1D	GSE33440
*VGLL4*	Hypo	3:11,610,728–11,610,728	-,-	shores,prom,intron	Control down vs T1D clinical onset	E-MEXP-1140
*CCT7*	Hypo	2:73,465,970–73,465,970	-,-	intron	Control down vs T1D clinical onset	E-MEXP-1140
					Control down vs T1D	GSE33440
*RANBP3*	Hyper	19:5,962,749–5,962,749	-,-	intron	Control down vs T1D clinical onset	E-MEXP-1140
					Control down vs T1D	GSE33440
*OLFM2*	Hypo	19:10,027,408–10,027,408	-,-	shores,intron	Control up vs T1D clinical onset	E-MEXP-1140
*TGIF1*	Hyper	18:3,412,523–3,412,523	-,-	prom,intron	Control down vs T1D	GSE33440
*PRPSAP1*	Hypo	17:74,350,980–74,350,980	-,-	shores,prom	Control up vs T1D	GSE33440
*ZNF778*	Hypo	16:89,300,215–89,300,215	-,-	shores,exon	Control up vs T1D clinical onset	E-MEXP-1140
*MMP26*	Hyper	11:5,012,592–5,012,592	11:5,012,001–5,013,000, 11:5,012,435–5,012,758	intron	Control up vs T1D	GSE43488
*YAP1*	Hypo	11:102,028,829–102,028,829	-,-	intron	Control up vs T1D clinical onset	E-MEXP-1140
*CNP*	Hyper	–	17:20,947,001–20,948,000,-	CpGi,shores,prom,exon,intron	Control down vs T1D clinical onset	E-MEXP-1140
*USP22*	Hyper	–	17:40,119,001–40,120,000,-	CpGi,shores,prom	Control up vs T1D recent onset	E-MEXP-1140
					Control up vs T1D 4 month after onset	GSE9006
*DRAM1*	Hyper	–	22:33,119,001–33,120,000, 22:33,119,131–33,119,362	CpGi,shores,prom	Control down vs T1D recent onset	E-MEXP-1140
*TIMP3*	Hypo	–	8:33,062,001–33,063,000, 8:33,062,053–33,062,289	intron	Control down vs T1D clinical onset	E-MEXP-1140

### Gene expression validation in extended cohort of familial T1D

**Figure 3:**
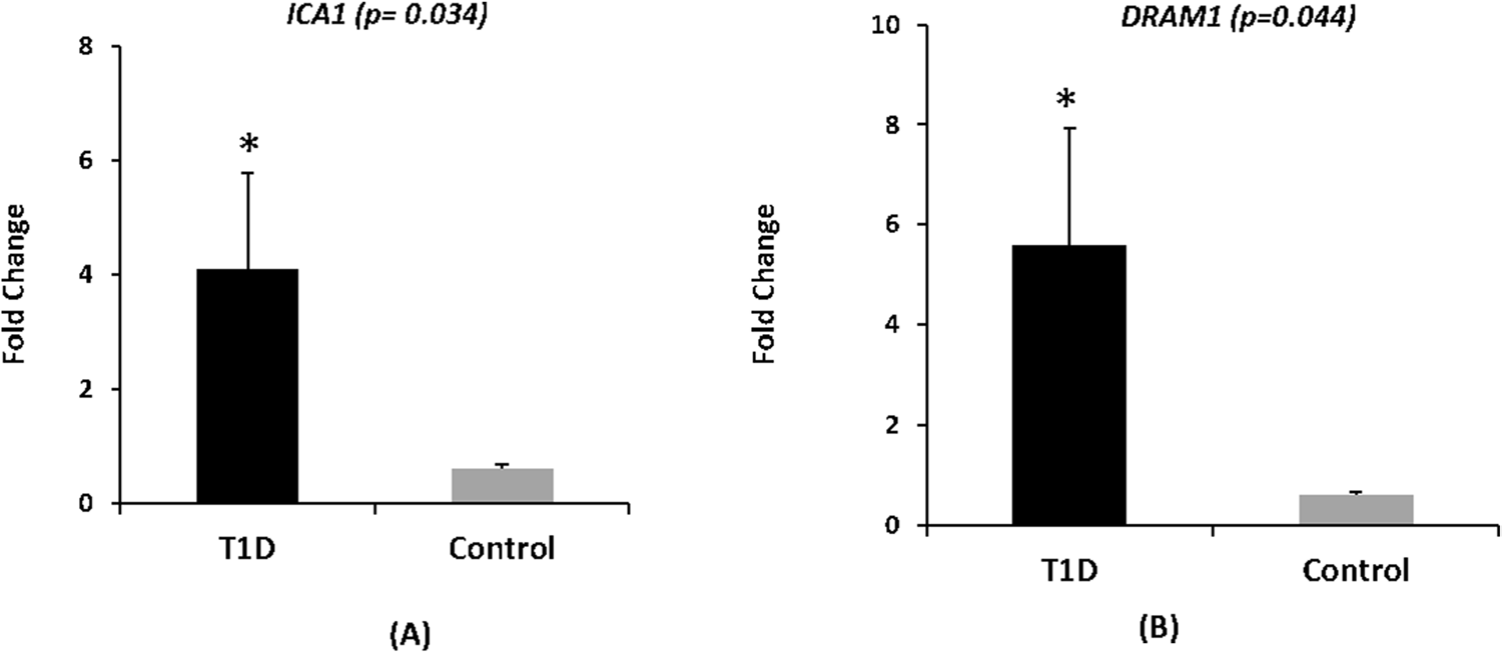
Bar plot indicating the expression level of (**A**) *ICA1* and (**B**) *DRAM1* in T1D versus control samples.

Quantitative real-time PCR was performed to validate two of the shortlisted candidate genes, namely *ICA1* and *DRAM1* (Fig. 3). These two genes were comparatively biologically more relevant to the pathophysiology of T1D. Analysis of individual families revealed an increase in the expression of *ICA1* (FC ≥ 2) in 12 of the 33 tested T1D families from the extended study cohort. Similarly, an increased expression of *DRAM1* (FC ≥ 2) was observed in 10 of the 33 tested T1D families. Compiled analysis revealed a significantly increased expression of *ICA1* (*p* = 0.034) and *DRAM1* (*p* = 0.044) in individuals afflicted with T1D compared to their unaffected family members.

## Discussion

This study conducted methylation profiling of three HLA-identical T1D families in order to explore epigenetic contribution to the onset of T1D. Exploring methylation differences between the affected and unaffected individuals from twins and families minimize variation due to environment or genes and reduce residual noise variance due to factors such as population stratification. An integrative approach involving relevant human transcriptomics data that accelerated the prioritization of candidate genes for T1D was employed.

The PCA of methylation profiling of single CpG sites across the three T1D families demonstrated a familywise clustering of affected individuals, which might suggest a common, but family-specific methylation pattern in T1D. Both the monozygotic and dizygotic twins showed a tighter cluster in PCA analysis as opposed to that seen with non-twinned siblings, suggesting a similar methylation pattern in twins; thus, such a design is better suited as it helps to reduce spurious differences in methylation events because of confounders. Moreover, a higher degree of closeness could be observed between the monozygotic twins in family B than in the dizygotic twins in family A. This observation adheres to the finding of Hannon et al*.*^13^ who demonstrated that monozygotic twins have higher correlation of site-specific levels of DNAm compared to dizygotic twins.

Differential DNAm events were more prominent within intergenic regions and genes, compared to regulatory regions such as promoters, CpG islands, and CpG shores. Similar observations were reported by a study from the Encyclopedia of DNA Elements project that investigated the dynamics of DNAm patterns and relationships to regulatory elements^14^. Furthermore, the observed high percentages of differentially methylated events at promoter regions compared to exons may suggest that there is capture enrichment for promoters by the methylation kits target design^15^. Although the pathway enrichment analysis revealed the T1D pathway, it was not statistically significant going by FDR value. The inability to identify any significantly enriched pathway is possibly because of the small size of the inputted gene list.

The genomic location of DMC and DMR is crucial in the up-/downregulation of gene expression. Moreover, methylation over CpG functional regions (such as CpG island of around 1 Kb in length)—often located near or in the gene promoter region close to the TSS and/or CpG island shore (within 2 Kb from islands) near promoters—may reduce or inhibit gene expression^16^, while methylation at sites within a gene, such as the region after the first exon, may result in an increase in gene expression^16^.

This study prioritized 18 genes by comparing a set of 84 differentially methylated genes with a published set of differentially expressed genes in T1D. Literature reports supported the role of some of these 18 genes in T1D as listed below:

*ICA1*: This gene is involved in membrane trafficking at the Golgi complex, including transport of insulin in immature secretory granules^17,18^. *ICA1* is an autoantigen in insulin-dependent diabetes mellitus^19–21^; however, it has a low number of detectable ICA in Chinese patients compared to Caucasians ^22^. The expression of *ICA1* is high in human pancreatic islets^23^ and is upregulated in pancreatic tissue at the clinical onset of T1D (Table 2). The identified hypermethylation events of *ICA1* at the gene body correlate with its upregulation expression in our T1D patients (Fig. 3), providing evidence for DNAm mediated regulation of gene expression. However, it is difficult to discern its causality until a fully integrated functional genomics strategy is performed^24^. Nevertheless, it is interesting that our study identifies differential methylation events in one of the four major autoantibodies (ICA, GAD, IAA, and IA-2A) that characterize T1D. It is interesting to note that Rakyan et al*.*^25^ observed T1D-associated methylation variable positions (T1D-MVPs) in one of these four autoantibodies, namely GAD2.

*MMP26*: This gene has been associated with higher fasting plasma glucose levels^26^ and impaired wound healing in diabetic patients because of its role in extracellular matrix (ECM) degradation^27^.

*TIMP3*: This gene is functionally related to ECM regulation. Our study identified this gene from both the tiles and targeted DMR analysis. It had been shown that *TIMP3* expression was decreased in the kidney of STZ treated-mice, a well-known model for hyperglycemia and glucotoxicity, reproducing T1D^28^.

*DRAM1*: We observed hypermethylation of this gene in DMR analysis in the CpGi and promoter regions. Hypermethylation of the CpGi and promoter regions is usually associated with gene silencing. Nevertheless, blood samples from our T1D patients revealed that it was upregulated (Fig. 3). Other research groups observed similar results after performing integrated analysis in which DNAm in functional regions was both concordantly and discordantly associated with gene expression^29,30^. Smith et al.^31^ demonstrated that increasing levels of promoter methylation do correlate directly with increased gene expression in a selection of contexts. It is possible to present a context-dependent model whereby epigenetic contributions to transcriptional regulation occur through diverse mechanisms in a more complex and dynamic manner^31,32^. Functionally, the loss of *DRAM1* has been demonstrated to inhibit the mechanistic target of rapamycin complex 1 (mTORC1) activation that affects insulin signaling, glycemic balance, and adipocyte differentiation^33^.

*USP22*: This gene suppresses high glucose-induced apoptosis in podocytes when silenced^34^.

*PIK3CG*: This gene is known to suppress autoimmune diabetes when suppressed^35^.

*EXT1*: The Exostosin Glycosyltransferase 1 gene encodes an enzyme that is critical for Heparan Sulfate synthesis in β-cells that regulate insulin secretion and glucose homeostasis^36^.

The identified DNA methylation events in our study have little in common with those reported in literature. Reasons for this disparity include the following: Apart from one that adapted MZ concordance for T1D^9^, reports from literature adapted twins that were T1D-discordant^8,25,37,38^. In addition, most of these DNAm studies were array-based which can have an impact on the overall results when compared to NGS-based DNAm profiling. For instance, the methylation profiling studies of Rakyan et al.^25^ and Stefan et al.^9^ revealed less than 133 CpG sites displaying significant methylation changes using Illumina HumanMethylation 27 K array. The methylation profiling studies on T1D of Disanto et al.^37^ and Elboudwarej et al.^38^ revealed more than 1,000 CpG sites using Illumina HumanMethylation 450 K array. Thus, the results can vary among the same array-based DNAm profiling studies. Moreover, we used HLA-identical (Arab) families, an approach which was used only in one of the above-listed previous studies namely that of Elboudwarej et al.^38^ on western population.

The present study has certain limitations. Firstly, we explored specific types of families having mono- or dizygotic twins concordant for T1D phenotype in order to identify novel candidate genes for T1D using NGS-based technology from a small size population in Kuwait. This made it difficult to have a large cohort size when compared to other array-based DNA methylation studies in the larger populations^8,10^. Secondly, gene expression profiles derived from blood samples from the three families in our study to integrate with DNA methylation events is probably not the best prioritization approach for T1D candidate genes. To compensate, we utilized (a) publicly available relevant transcriptomics data from blood and pancreatic tissue, and (b) larger cohort to validate the prioritized T1D candidate genes. Thirdly, peripheral blood sample is not the ideal biological sample as (a) it contains a diverse mixture of cells and DNAm is cell-specific^39^ (b) it may harbour DNA methylation events due to pathological differences relating to autoimmune processes that we could not statistically adjust for^10^. Additionally, Paul et al*.*^8^ demonstrated an increased DNAm variability in T1D across the three immune cells. Despite this, Disanto et al.^37^ observed a high correlation of methylation events in immune cell types between controls and T1D quadruplets, suggesting a common driving mechanism in more than one immune cell type. Furthermore, blood as biological sample has been used in other DNA methylation studies on T1D^10,37,40^ as well as in publicly available gene expression data used to prioritize T1D candidate genes in our study. Nevertheless, our future plans include use of enriched cell types from the peripheral blood to focus on identifying cell type-specific gene regulatory circuits involved in immune cell metabolism and the cell cycle.

## Conclusion

DNAm profiling of Arab familial T1D in Kuwait revealed that several genes are related to T1D. The integrative approach of using differential methylation events with numerous publicly available transcriptomics data allowed us to shortlist functionally relevant genes that can be differentially methylated in T1D pathogenesis. Validation of the candidate genes in an independent cohort of T1D families from the same ethnicity helped to identify *ICA1* and *DRAM1* as the most plausible candidates. Monozygotic twins are tightly clustered in the PCA scatter plot suggesting a similar methylation pattern. Finally, in order to discern the methylation events that cause or respond to the disease, a large cohort (of prediabetes or T1D versus healthy individuals) with gene expression profiling is needed to replicate the findings of our study.

## Materials and methods

### Ethics statement and study cohort

The Ethical Review Committee of Dasman Diabetes Institute reviewed and approved the study protocol as per the guidelines of the Declaration of Helsinki, and of the United States Federal Policy for the Protection of Human Subjects.

T1D families were recruited from the outpatient pediatric diabetes clinic at Dasman Diabetes Institute based on physician referrals from Ministry of Health, Kuwait. Three families in each of which at least two children (twinned or non-twinned) were diagnosed with T1D were considered in this study. The T1D diagnosis criteria used are detailed in^12^. In brief: Diagnosis was established according to WHO criteria^41^, which include fasting hyperglycemia and absolute insulin deficiency as measured by C-Peptide level. The date of first insulin injection was taken as the date of onset.

Prior to participating in the study, every adult participant signed the informed consent form. As regards the pediatric subjects, informed consent was obtained from the parents/legal guardians and assent was obtained from children aged 7 years or more.

### Blood sample collection and processing

Blood samples were collected in ethylenediaminetetraacetic acid (EDTA)-treated tubes and the genomic DNA was extracted using a QIAamp Blood DNA kit (Qiagen, Germany). A Qubit Fluorometer (Thermofisher, USA) was used to quantify spectrophotometry according to the manufacturer’s protocol. Absorbance values at 260–280 nm was checked for adherence to an optical density range of 1.8–2.1.

### Bisulfite sequencing using Illumina Truseq Methyl Captures EPIC kit

Genomic DNA (500 ng) was subjected to fragmentation using Covaris E220 (Covaris Inc, USA). Purified DNA fragments were then used with Truseq Methyl Capture EPIC Library Prep Kit reagents (Illumina Inc, USA) for library preparation, according to the Illumina protocol. The protocol involved adapters ligation, hybridization of the libraries with Illumina adapters, followed by bisulfite conversion and amplification. Additionally, KAPA HiFi HotStart Uracil + ReadyMix Kits (Roche, Switzerland) were used to enrich the resultant library. The enriched libraries were later pooled and multiplexed for up to six samples and clustered by Truseq Paired Cluster Kit V3 (Illumina Inc, USA). Paired-end sequencing was performed using the Illumina HiSeq 2500 sequencing platform.

### DNA methylation (DNAm) bioinformatics analysis

Raw Illumina paired-end bisulfite treated reads were *converted* into *FastQ format* by executing bcl2fastq2 Conversion Software version 2.20 (Illumina, USA) with the option of enabling sequence adaptors trimming. The quality of the sequenced data from FastQ files was assessed using FastQC version 0.11.9 (Babraham Bioinformatics, UK). Low quality reads and the first six base pairs of both the forward and reverse reads, were trimmed using *Trim Galore!* version 0.3.3 (Babraham Bioinformatics, UK), which utilizes Cutadapt version 1.1^42^. The trimmed reads were then aligned to in silico bisulfite-converted human genome hg19 using Bismark version 0.22.3^43^ and Bowtie2 version 2.3.5.1^44^. The resultant outputs of sequence alignment/map (SAM) files were subsequently sorted and were not subjected to a deduplication step as recommended for targeted capture sequencing.

SAM files were imported to the R console version 3.6.2 (https://www.R-project.org/) and were processed using methylKit package version 1.2.4^45^ to make methylated cytosine calling and to derive statistics on coverage of CpG bases, on per base methylation statistics, as well as to perform clustering and differential methylation calculations. CpG sites with a minimum read quality of at least 20 and a minimum read coverage of at least 10, as well as a maximum coverage of 99.9%, were considered for differential methylation analysis. In the single base CpG resolution analysis, methylation calls were optimized by combining methylated sites from both the forward and reverse reads using the destrand = TRUE option. For target region CpG resolution analysis (called as truseq analysis), we used the target regions of epigenetic interests as downloaded from the manifest file of the Illumina Truseq Methyl Capture EPIC kit. Tiles analysis was performed with a tiling window of 1000 bp. Clustering analysis based on levels of CpG methylation at base-pair resolution was performed on all the samples using Ward’s minimum variance method. Differentially methylated single CpG site (DMC) and regions (DMR) were detected if the methylation difference level between the compared groups was at least 25%, and methylKit q-value (equivalent to false discovery rate adjusted p-value) was ≤ 0.01. Genome annotations of the DMC and DMR regions (from both the truseq and tiles analyses) were performed using the methylKit package and the hg19 refseq files on genes and CpG islands as downloaded from the UCSC genome browser (http://genome.ucsc.edu/UCSC). Additionally, annotation of CpG sites—performed using genomation^45^ tool from R package—defined promoters as 1 kilo base (kb) upstream of transcription start sites (TSS), and CpG shores as 2 kb flanking CpG islands.

Environmental factors such as lifestyle, diet, and use of medication often correlate with disease status and influence epigenetic mechanisms^46^. Hence, exploring methylation differences between affected and unaffected individuals from twins and families can provide an estimation of contribution from such plausible factors to methylome variation and reduce residual noise variance. To understand this, unsupervised principal component analysis (PCA) and Hierarchical clustering analysis (HCA) with Ward’s hierarchical clustering approach were performed using functions available from the methylKit package.

### Pathway enrichment analyses

WEB-based GEne SeT AnaLysis Toolkit (WebGestalt;^47^) was used to perform pathway analysis through the method of over-representation analysis based on Kyoto Encyclopedia of Genes and Genomes (KEGG) that integrates genomic, chemical, and systemic functional information^48^. A *p*-value < 0.05 was used as a cut-off for false discovery rate (FDR) to detect significantly enriched pathways.

### Publicly available gene expression datasets for T1D

Gene Expression Omnibus and ArrayExpress databases were searched for published gene expression datasets on T1D by using “type 1 diabetes” as keyword. Genes that are differentially expressed with statistical significance were analyzed using the LIMMA package^49^ with a value of < 0.05 as threshold for FDR *p*-value (while adjusting for covariates when available). Significant differentially expressed genes were integrated with DNAm results to prioritize candidate genes for T1D.

### Validation of differentially expressed genes in an extended cohort of T1D families

Gene expression analysis was performed in an extended cohort of 33 T1D families including families A and B consisting of 63 affected and 65 unaffected family members. This cohort was collected as part of another study using similar ethical guidelines and recruitment protocols. RNA was extracted from peripheral blood using Qiagen RNA blood mini kit (Qiagen, Hilden, Germany) and then reverse transcribed using ABI reverse transcriptase kit (Applied Biosystem, USA) according to the manufacturer’s protocol. Quantitative real-time PCR was performed using pre-designed ready to use Taqman gene expression assays obtained from Applied Biosystem (Life technologies, NY, USA). Assays were selected based on maximum transcript variant detection: *ICA1* (#Hs00245256: NM_001136020.2, NM_001276478.1, NM_004968.3, NM_022307.2) and *DRAM1* (#HS00218048: NM_018370.2, XM_005269004.2, XM_017019578.1). Gene expression analysis of each target was individually carried out relative to *GAPDH* as endogenous control on ABI 7500 real time PCR system (Life technologies, NY, USA) following manufacturer’s protocol. The fold change (FC) for each sample relative to the control sample was calculated using the 2 − ΔΔCT method, with a cut-off value of ≥ 2. Differences in the expression level of candidate markers between the two tested groups were characterized using unpaired t-test.

## Supplementary Information

Supplementary Information 1.

Supplementary Information 2.

Supplementary Information 3.

Supplementary Information 4.

Supplementary Information 5.

Supplementary Information 6.

Supplementary Information 7.

Supplementary Information 8.

## Data Availability

The DNA methylation NGS data generated is deposited at the NCBI Sequence Read Archive (BioProject ID PRJNA783490) and will be released when the manuscript is accepted for publication.
