# Supplementary figures and images for: Differentially methylated and expressed genes in familial type 1 diabetes
Source: Sci Rep. 2022 Jun 30;12:11045. doi: 10.1038/s41598-022-15304-5 (PMC9247163; doi:10.1038/s41598-022-15304-5)

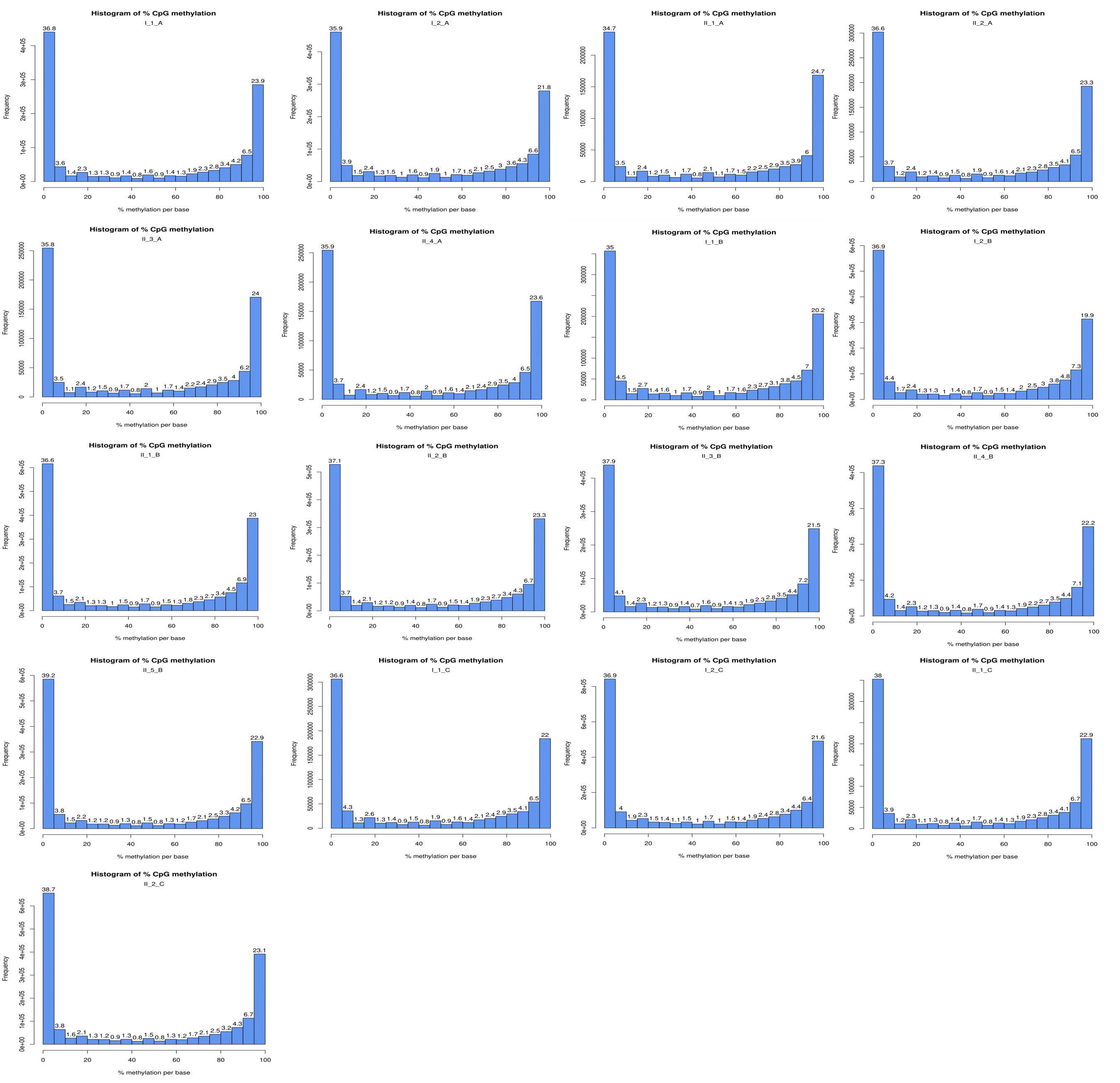

Supplement: Supplementary file 1 — Supplementary Information 1. [file 41598_2022_15304_MOESM1_ESM.jpeg]

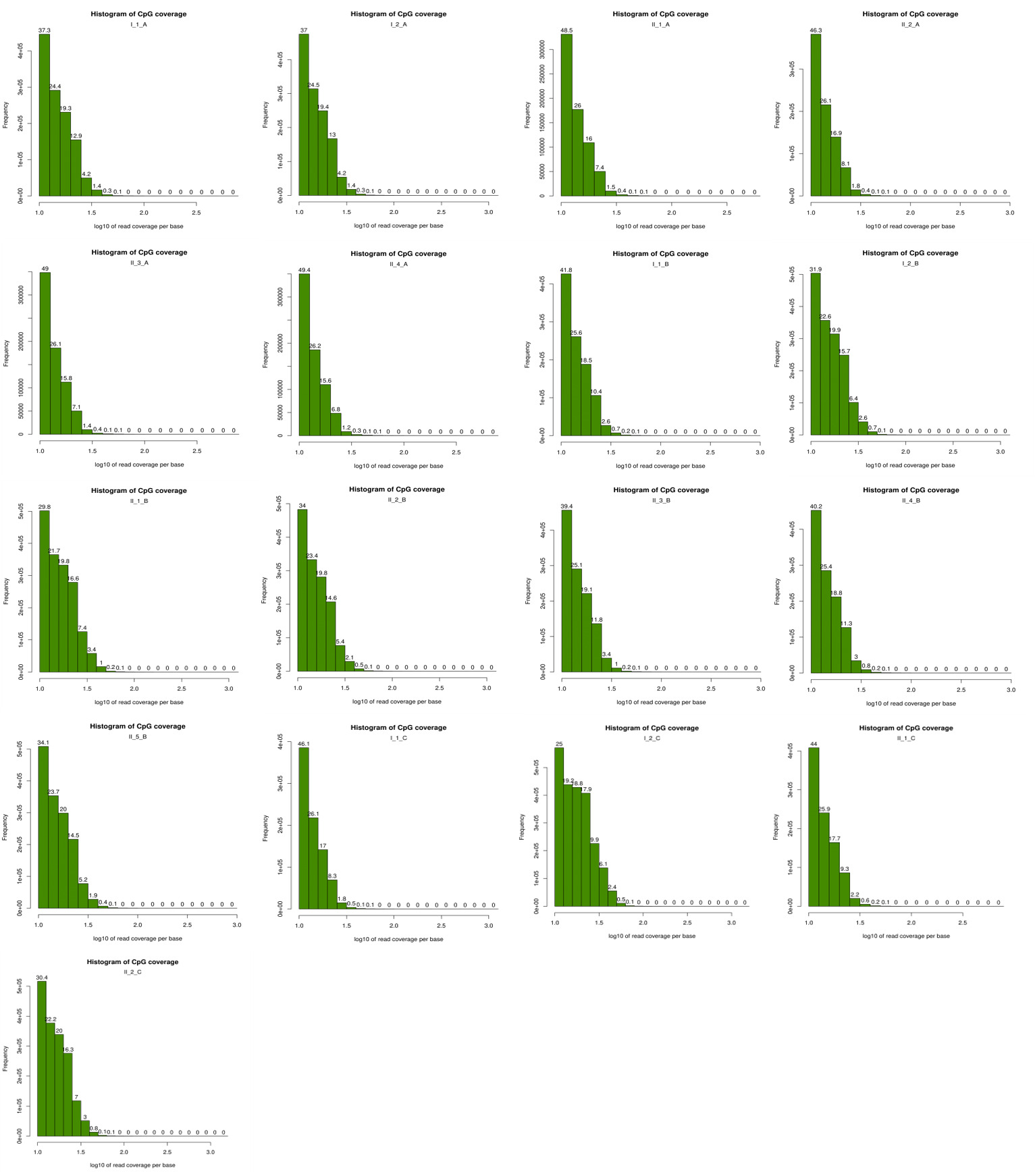

Supplement: Supplementary file 2 — Supplementary Information 2. [file 41598_2022_15304_MOESM2_ESM.jpeg]

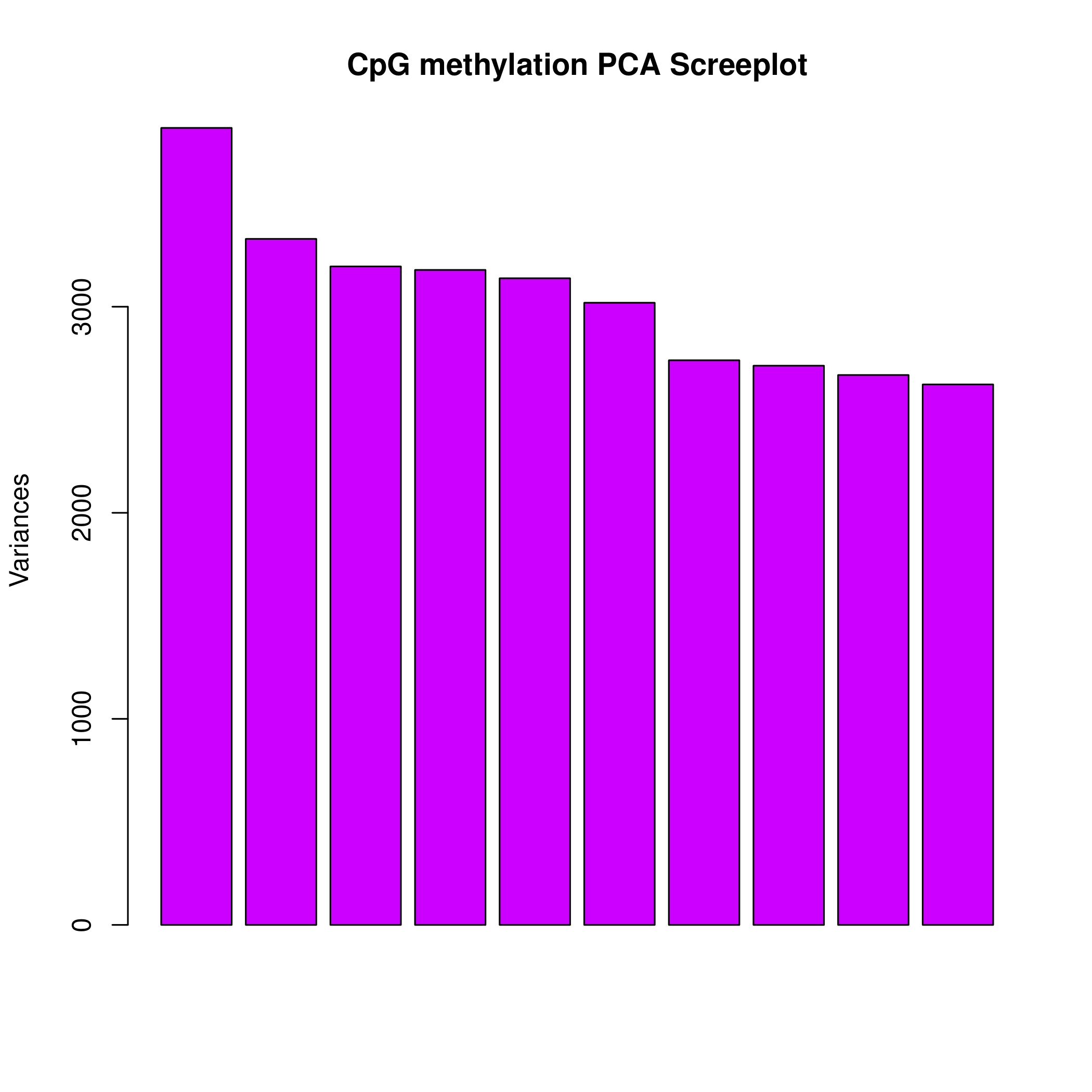

Supplement: Supplementary file 3 — Supplementary Information 3. [file 41598_2022_15304_MOESM3_ESM.jpeg]

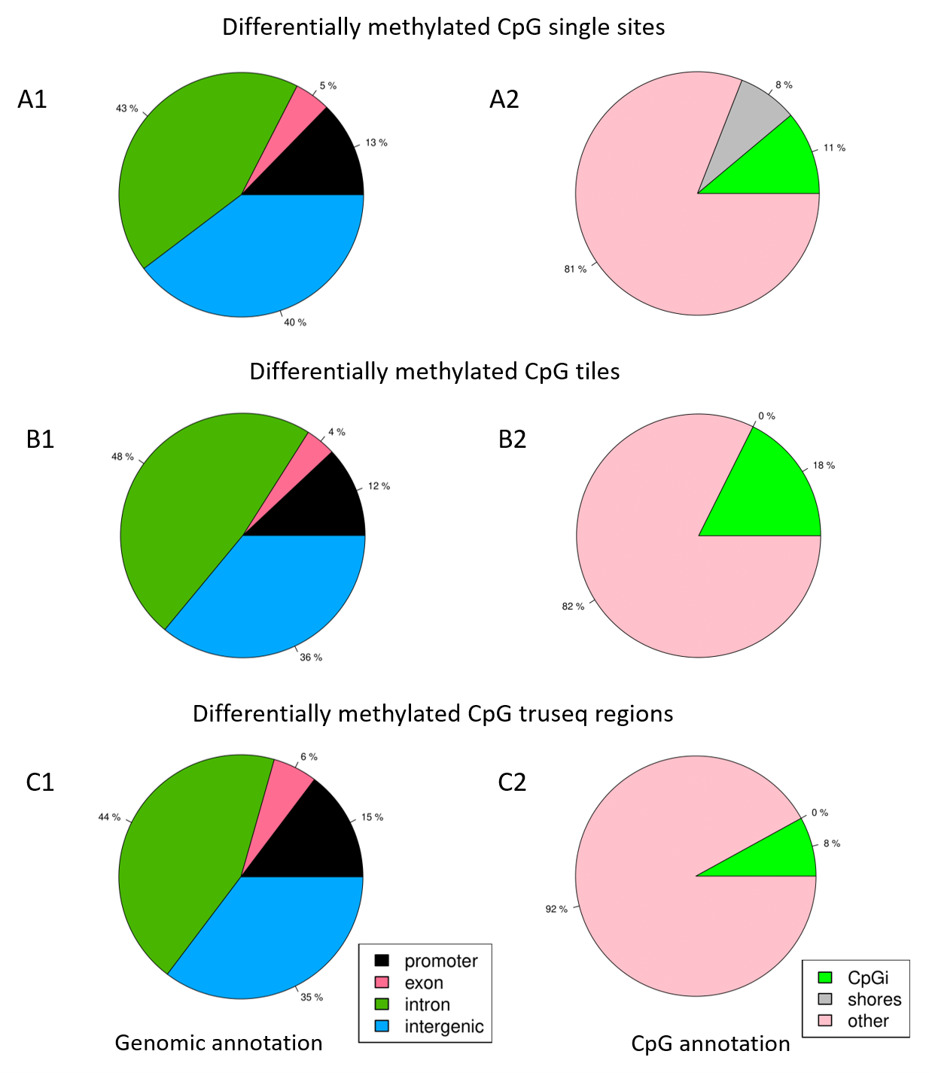

Supplement: Supplementary file 4 — Supplementary Information 4. [file 41598_2022_15304_MOESM4_ESM.jpeg]
