# Supplementary material for: Differentially methylated and expressed genes in familial type 1 diabetes
Source: Sci Rep. 2022 Jun 30;12:11045. doi: 10.1038/s41598-022-15304-5 (PMC9247163; doi:10.1038/s41598-022-15304-5)
Supplement: Supplementary file 5 — Supplementary Information 5. [file 41598_2022_15304_MOESM5_ESM.docx]

**SUPPLEMENTARY INFORMATION**

**Supplementary information**

**A. Exome sequencing of twins and estimation of zygosity among twins**

*A.1.* ***Exome sequencing of twins***

DNA samples of the twins from each family were enriched for whole exome sequencing using Nextera Rapid Capture Exome kit (Illumina Inc. USA) to genetically confirm their zygosity. Clustering of the captured libraries was carried out using TruSeq Paired-End Cluster Kit V3 (Illumina Inc., USA) and were later sequenced on HiSeq 2000 Illumina platform. Furthermore, extracted raw paired-end 100 bp reads were mapped to the human genome reference (GRCh37) using Burrows-Wheeler Aligner using default parameters (1). Aligned reads were converted to compressed binary format (BAM) files using SAMtools (2) followed by the removal of duplicate reads via Picard tool version 2.20.2 (<http://broadinstitute.github.io/picard>). Genome Analysis Tool Kit (GATK) version v3.8-1-0 (3) was used to estimate exome coverage as well as to perform variants calling.

***A.2. Estimation of zygosity among twins***

Total heterozygous SNPs identified from the dizygotic twins in family A were 17,249 and 17,063 in which 10,185 (42%) were seen to be in common. The total extracted heterozygous SNPs of the monozygotic twins from family B were 17,286 and 17,266 in which 16,573 (92%) were found to be in common. Furthermore, total homozygous variants for the dizygotic twins in family A were 9,430 and 9,347 in which 7,081 (76%) were in common. Total homozygous variants of the monozygotic twins in family B were 11,214 and 11,221 in which 11,060 variants (97%) were in common.

**B. HLA typing profile of DRB1, DQA1 and DQB1 alleles in T1D patients**

Raw FastQ files of T1D patients from the three families (A, B and C) were used as input for the tool of HLA -HD Version 1.4.0 (http://www.genome.med.kyoto-u.ac.jp/HLA-HD) to perform HLA typing. The IPD-IMGT/HLA database version 3.46 (2021-October) build 2d19adf (https://www.ebi.ac.uk/ipd/imgt/hla/) was used for HLA typing.

**C. DNAm alignment and quality control results**

The number of aligned paired-end reads of an average sequence length of 70 bp with a unique best hit to the human genome reference was found to be about 11 million reads, which is more than 85% of the total sequence pairs. In total, a conversion rate of at least 99.6% of all non-CpG cytosine positions being converted to uracil and an average 45% of methylation CpG across all the samples were seen. This pattern is consistent across all the samples demonstrating that there is no significance difference between affected and unaffected individuals (see Supplementary Figure S1). Similarly, the distribution of methylation read coverage per base across all the samples was seen as bimodal indicating that the experiment did not experience PCR duplication bias (see Supplementary Figure S2).

**References**

1. Li, H. & Durbin, R. Fast and accurate short read alignment with Burrows-Wheeler transform. *Bioinformatics* **25**, 1754–1760. <https://doi.org/10.1093/bioinformatics/btp324> (2009).
2. Li, H. et al. The sequence alignment/Map format and SAMtools. *Bioinformatics* **25**, 2078–2079. <https://doi.org/10.1093/bioinformatics/btp352> (2009).
3. McKenna, A. et al. The genome analysis toolkit: A MapReduce framework for analyzing next-generation DNA sequencing data. *Genome Res.* **20**, 1297–1303. <https://doi.org/10.1101/gr.107524.110> (2010).

**Supplementary figures**

**Supplementary Figure S1. Percentage CpG methylation distribution across all the samples**. Histograms showing the overall percentage methylation showing two peaks at both ends of the plot representing that most of the bases are either methylated or unmethylated. This pattern is consistent across all the samples demonstrating that there is no significance difference between affected and unaffected individuals.

**Supplementary Figure S2. Read coverage per base of CpG sites distribution across all the samples.** Histograms of the overall read coverage per base of CpG sites showing one peak toward the left indicating most of the bases either have high or low methylation as well as the experiment did not suffer from PCR duplication bias.

**Supplementary Figure S3: Scree plot displays variance for the first 10 principal components.**

**Supplementary Figure S4. Differentially methylated CpG annotation across all familial T1D.** Pie charts showing percentages of differentially methylated CpG single sites (A), tiles (B), truseq regions (C) across genomic (1) and CpG (2) features. Methylated events are more prevalent in intronic and intergenic regions outside of CpG islands and shores.

**Supplementary tables**

**Supplementary Table S1.** Differential methylation events identified across the three T1D families.

**Supplementary Table S2.** The 84 unique differentially methylated genes across the three familial T1D.

**Supplementary Table S3.** Differentially expressed genes of published T1D gene expression data.
